# Understanding real‐world clinical experience with lecanemab: Capturing the patient and care partner voice through social media listening

**DOI:** 10.1002/alz70860_102018

**Published:** 2025-12-23

**Authors:** Monica K Crane, Lee Watson, Amanda Blackwood, Pippa Brown, Vicky Britton, Alison Bulkley, Marc DeCongelio, Craig Plauschinat, Feride H Frech, Hideki Toyosaki

**Affiliations:** ^1^ Genesis Neuroscience Clinic LLC, Knoxville, TN, USA; ^2^ University of Tennessee‐Knoxville, Knoxville, TN, USA; ^3^ Genesis Neuroscience Clinic, Knoxville, TN, USA; ^4^ Oracle Life Sciences, London, United Kingdom; ^5^ Oracle Life Sciences, Austin, TX, USA; ^6^ Eisai, Inc, Nutley, NJ, USA; ^7^ Eisai Inc., Nutley, NJ, USA

## Abstract

**Background:**

There is limited evidence of real‐world clinical experience with lecanemab from a patient and care partner perspective. The objective of this study was to better understand the direct clinical experiences of Alzheimer's Disease (AD) patients and care partners with lecanemab using social media posts.

**Method:**

This retrospective study examined lecanemab‐specific public social media data from AD patients and care partners based in the U.S. between January 2023 and October 2024. Using Brandwatch, Sprinklr and Buzzsumo, 468 lecanemab‐related posts were collected via detailed keyword strings which identified posts using first‐person or familial relation‐related language. Of these posts, 78 showed direct clinical experience with lecanemab and were assessed to understand treatment experience. Key themes were identified qualitatively through a systematic review by two researchers, with a third overseeing the process to ensure rigor and reduce bias. Quantitative insights were based off keyword frequency.

**Result:**

Qualitative data was retrieved from Reddit (58 posts, 74%), X.com (9 posts, 12%), digital media (8 posts, 10%), and Facebook (3 posts, 4%). Care partners indicated that they felt lecanemab slowed disease progression (*N* = 13), allowing them to spend more quality time with their loved ones. Treatment expectations for lecanemab influenced sentiments towards clinical outcomes. Care partners shared positive experiences with lecanemab contributing to cognitive and functional improvements in daily activities (*N* = 16), such as better cognitive test results, improved walking and balance, enhanced visual skills, and ability to cook. Inability to unequivocally recognize treatment success was expressed with frustration by care partners. “Side effect” was mentioned (*N* = 30), including Amyloid‐related imaging abnormalities “ARIA” (*N* = 13), “brain bleed” (*n* = 6), and “brain swelling” (*N* = 5). Qualitative social media data suggests that side effects are an important consideration in the treatment selection process.

**Conclusion:**

Qualitative social media themes described lecanemab as slowing disease progression and extending patients’ quality time with their loved ones. The posts report side effects which may influence beginning or continuing treatment. Although currently a small sample, social media listening may reveal considerations and opportunities to improve patient care.

